# Discriminating between sick and healthy faces based on early sickness cues: an exploratory analysis of sex differences

**DOI:** 10.1093/emph/eoad032

**Published:** 2023-09-27

**Authors:** Arnaud Tognetti, Evelina Thunell, Marta Zakrzewska, Jonas Olofsson, Mats Lekander, John Axelsson, Mats J Olsson

**Affiliations:** Department of Clinical Neuroscience, Karolinska Institutet, Stockholm, Sweden; CEE-M, CNRS, INRAE, Institut Agro, University of Montpellier, Montpellier, France; Department of Clinical Neuroscience, Karolinska Institutet, Stockholm, Sweden; Department of Clinical Neuroscience, Karolinska Institutet, Stockholm, Sweden; Department of Psychology, Stockholm University, Stockholm, Sweden; Department of Clinical Neuroscience, Karolinska Institutet, Stockholm, Sweden; Department of Psychology, Stress Research Institute, Stockholm University, Stockholm, Sweden; Osher Center for Integrative Health, Karolinska Institutet, Stockholm, Sweden; Department of Clinical Neuroscience, Karolinska Institutet, Stockholm, Sweden; Department of Psychology, Stress Research Institute, Stockholm University, Stockholm, Sweden; Department of Clinical Neuroscience, Karolinska Institutet, Stockholm, Sweden

**Keywords:** sex differences, sickness detection, behavioral immune system, disease-related personality traits, facial cues of sickness

## Abstract

**Background and objectives:**

It has been argued that sex and disease-related traits should influence how observers respond to sensory sickness cues. In fact, there is evidence that humans can detect sensory cues related to infection in others, but lack of power from earlier studies prevents any firm conclusion regarding whether perception of sickness cues is associated with sex and disease-related personality traits. Here, we tested whether women (relative to men), individuals with poorer self-reported health, and who are more sensitive to disgust, vulnerable to disease, and concerned about their health, overestimate the presence of, and/or are better at detecting sickness cues.

**Methodology:**

In a large online study, 343 women and 340 men were instructed to identify the sick faces from a series of sick and healthy photographs of volunteers with an induced acute experimental inflammation. Participants also completed several disease-related questionnaires.

**Results:**

While both men and women could discriminate between sick and healthy individuals above chance level, exploratory analyses revealed that women outperformed men in accuracy and speed of discrimination. Furthermore, we demonstrated that higher disgust sensitivity to body odors is associated with a more liberal decision criterion for categorizing faces as sick.

**Conclusion:**

Our findings give strong support for the human ability to discriminate between sick and healthy individuals based on early facial cues of sickness and suggest that women are significantly, although only slightly, better at this task. If this finding is replicated, future studies should determine whether women’s better performance is related to increased avoidance of sick individuals.

## 1. Introduction

While human mortality rates from infectious diseases have decreased globally over time, infectious disease remains a major source of mortality worldwide. In fact, fatal epidemics (e.g. influenza, ebola, and coronavirus disease 2019) are expected to increase in occurrence with climate change and globalization [1]. Thus, determining the behavioral mechanisms that prevent interpersonal contagion could guide public health interventions focused on infectious-disease transmission.

Due to their long coexistence with pathogens, humans and other extant species have evolved sophisticated immune systems to defend against infectious agents. Still, infections are debilitating and sometimes fatal [2]. One way to stay healthy is to avoid contact with pathogens, and many species engage in a wide range of prophylactic behaviors against infections [3]. This behavioral defense against infection includes perceptual mechanisms for detecting cues of infectious pathogens which, in turn, trigger disgust (a basic emotion which facilitates avoidance of infectious disease and toxins), and behavioral avoidance [4]. Still, our current understanding of the human behavioral repertoire that reduces the risk of infection remains limited. Notably, humankind is an inherently social species and avoidance of infected individuals may be costly as it deprives individuals from valuable social interactions such as mating opportunities, social learning, childcare or foraging. Hence, pathogen avoidance behaviors should only be adopted when their expected benefits outweigh their expected costs. However, the characteristics that influence how an individual respond to pathogens remains to be determined.

Many social species, including mandrills, mice, and lobsters, identify sick conspecifics via olfactory and visual cues and strategically avoid physical contact with them [3, 5–7]. Humans are likewise able to identify sick individuals based on cues of sickness, such as vomiting, coughing, sneezing or rashes, and following exposure to these cues, individuals likely feel disgust and engage in behavioral avoidance (e.g. increase their interpersonal distance to the sick conspecific) [8, 9]. However, these aforementioned sickness cues, are typically expressed when people have been contagious for a while and at the later stages of infection. The ability to detect and avoid sick individuals in early phases of an infection would be even more efficient in reducing the likelihood of contamination.

A growing body of research suggests that humans can detect cues characterizing the early phases of sickness. For instance, a series of studies that experimentally induce sickness via injection with an endotoxin (lipopolysaccharide, LPS) showed that sickness can be identified above chance already a couple of hours after injection based on faces, body odors and biological motion [10–14]. Skin coloration is likely a reliable cue of early sickness as naïve observers perceive sick volunteers as paler in their skin and lip color compared to the same volunteers when healthy [10]. In fact, this change in skin color is objectively measurable as early as 1 h after injection with endotoxin [15]. Furthermore, sick volunteers exhibit more hanging eyelids and drooping corners of the mouth and are rated as less attractive, less healthy and expressive of more negative emotions compared to healthy faces [10, 12, 16]. Finally, there is evidence that sick volunteers are less liked, suggesting that cues of sickness may trigger adaptive social responses, such as avoidance [12, 13].

Despite the evidence that sickness cues can be perceived by observers and that such perception leads to attitudes that may precede disease-avoiding behaviors, little is known about inter-individual variation in the detection of these cues, or how this variation influences the propensity for behavioral avoidance. There is evidence that some personality traits are related to individual reactions to disease-related stimuli. For example, individuals with higher perceived vulnerability to disease (PVD) seem to be more alert towards conspecifics who display a sickness cue, or towards objects that could potentially transmit an infection, and upon detection, they may exhibit stronger avoidance behaviors [4, 17]. Moreover, individuals suffering from health anxiety, a persistent fear of being or falling ill, perceive others as less healthy, and also rate the risk of contagion as greater compared to individuals with low levels of health anxiety [18]. In parallel, individuals that are easily disgusted tend to exhibit more behavioral avoidance of sickness-connoting stimuli [19]. To the best of our knowledge, a few studies have examined the influence of such personality traits on individual reactions to early cues of sickness, none of which found any associations [13, 20]. However, these studies used relatively small sample sizes (*N* = 77 and 44, respectively), preventing any firm conclusions.

Sickness detection and behavioral avoidance may differ between men and women. While parental investment is a crucial resource for human children, paternal investment is facultative and shows larger inter-individual variation compared to maternal investment [21, 22]. For example, evidence from (mostly) natural fertility populations suggests that while the presence of a mother is pivotal for child survival, fathers have little effect on child survival [22]. Hence, women may be more likely to avoid sick conspecifics than men, because they have a more central role in protecting themselves and offspring from disease [9, 23]. Moreover, as women are more susceptible to sexually transmitted infections, heightened perception and response to sickness cues may be advantageous, in reducing their infection risk [23, 24]. As such, women may display a greater accuracy—and a lower bias—in detecting disease-relevant cues, or, alternatively, an increased likelihood in falsely identifying noninfectious individuals as infectious (i.e. a higher bias). In line with this, women report higher disgust sensitivity when exposed to conspicuous disease-related stimuli [9, 25, 26] and exhibit higher scores on the Disgust Scale, a scale sampling seven domains of disgust elicitors [27, 28], as compared to men. However, whether women are more accurate (i.e. less biased) or less accurate (i.e. more biased) in detecting early and more subtle cues of sickness remains unknown.

In the present study, we tested whether humans can discriminate between ‘healthy’ and ‘sick’ faces, and whether the perception of cues of sickness is associated with sex, self-reported health and disease-related personality traits, by using signal detection methodology that enables the separation of detection accuracy from the behavioral tendency (bias) to categorize stimuli as disease cues [29]. We used a unique set of facial photographs from an LPS injection study. The set consists of pairs of photographs for a number of individuals: one taken in a healthy control condition, and the other in an experimental sickness condition. In a large online study, we presented these facial photographs to participants (*N* = 683) sequentially and asked them to identify each face as either sick or healthy. They also completed several disease-related questionnaires. Based on previous studies [10, 12, 13], we predicted that humans would be better than chance at discriminating between healthy and sick individuals based solely on early facial cues of sickness. We also predicted that perception of disease cues would differ between men and women, with women being more biased—that is more likely to overestimate the presence of sickness cues in a face. In addition, we explored if women were worse or better at differentiating between sick and healthy faces compared to men. Finally, we predicted that individuals who are more sensitive to disgust, vulnerable to disease, concerned about their health and self-report a poorer health, would also be more likely to overestimate the presence of facial sickness cues.

## 2. Methods

The design and analysis plan were preregistered on OSF prior to data collection (https://doi.org/10.17605/OSF.IO/P85EZ).

### 2.1. Facial stimuli

#### 2.1.1 Collection

The facial stimuli (photographs) were collected in a previous study investigating sickness detection and behavioral and physiological changes during acute systemic inflammation. A more detailed description of the experimental model and stimuli acquisition is available in [10, 30]. The initial study, as well as other studies that used the stimuli, were approved by the regional ethical review board in Stockholm, Sweden (Dnr 2014/1946-31/1). In short, twenty-two healthy volunteers (mean age 23 years; nine women) participated in a within-subjects, double-blind and placebo-controlled study using the experimental endotoxemia model. These participants, hereafter called donors, were randomly assigned to either first receive a LPS injection (Escherichia coli endotoxin, Lot HOK354, CAT number 1235503, United States Pharmacopeia, Rockville, MD, USA) at 2.0 ng/kg body weight or a placebo injection (0.9% NaCl), and the reverse treatment approximately 1 month later. In both conditions (LPS and Placebo), pro-inflammatory cytokines, subjective sickness ratings, and tympanic temperature were measured to confirm that a significant inflammatory response was caused by the LPS administration (for more details see [30]). Facial photographs of the donors were taken 2 h after injection, which corresponds, in the LPS condition, to the approximate peak of the pro-inflammatory cytokine levels [10, 30]. The facial photographs were obtained using a Nikon D90 (Nikon Corp., Tokyo, Japan), with a focal length of 50 mm (a 50 mm lens was used), aperture speed at 1/125, and 200 ISO and the same lighting conditions. All donors wore identical white t-shirts, were asked to sit comfortably, look straight into the camera, and express a neutral facial expression, and were restricted from wearing any make-up.

#### 2.1.2 Selection and preparation of facial stimuli

**Figure 1.**
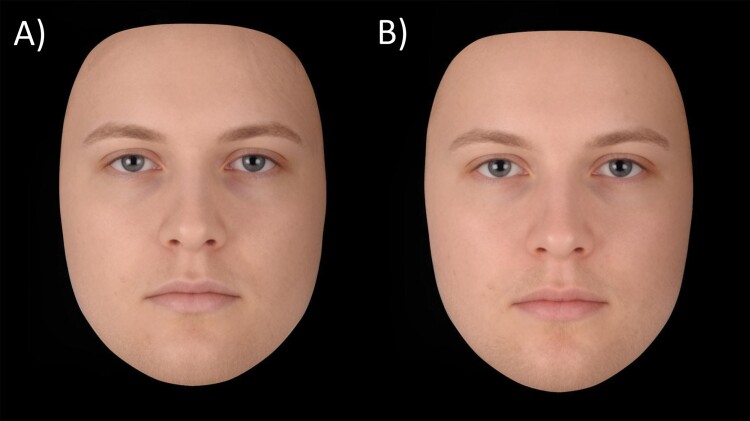
Facial composites displaying the average shape, color and texture of 20 donors (eight women) photographed twice in a cross-over design, during experimentally induced (A) acute sickness and (B) placebo.

To prevent recognition of donors, and circulation of the donors’ faces on the internet, we created realistic virtual composite faces based on the donors facial photographs that kept sickness cues intact [31]. All composite faces were made with *Webmorph*, a web-based version of *Psychomorph* and this involved three steps [32]. First, same-sex donors were randomly assigned to pairs. Second, the two paired faces were combined within a given condition (LPS or Placebo) resulting in composites presenting a 50:50 combination of shape, color and texture information from both original faces. Third, each composite was cropped to include the face only and the background was changed to black. This procedure resulted in a new dataset of 20 facial composites (10 healthy and 10 sick) from six virtual men and four virtual women (for an illustration of our facial stimuli, please refer to Fig. 1, which depicts a composite of the faces of the 20 donors in each condition.) Out of the 22 donors, one man and one woman were excluded because their faces were too obscured by hair, making the composites appear unrealistic.

### 2.2 Main experiment

#### 2.2.1 Participants

We collected full data sets from 343 women (mean age = 34.30 years, SD = 12.18, range = 18–72 years) and 340 men (mean age = 35.29, SD = 12.95, range = 18–75 years). Age distribution of the participants is presented in Supplementary Fig. S1. Another 32 participants started the study but were excluded because they failed the quality check questions (see below). Our sample exceeded our initial aim (*N* = 680), which was based on a power analysis allowing for detection of a weak association between personality traits and perception of facial cues of sickness (*r* = 0.15) with a power of 80% and an alpha level = 0.001 for a planned directional hypothesis, or 80% power to detect a sex difference as small as *d* = 0.22 with an alpha level of 0.05 (two-directional hypothesis). All participants were recruited using the online recruitment system *Prolific* (https://www.prolific.co). Participants whose first language was English (the language used during the experimental procedure), and who identified as either male or female (both biological sex and gender) according to their prolific profile were invited to the study. All participants received a flat fee of £2.50 (currency used by the *Prolific* platform) as compensation for their participation. They completed the study in 11 (SD = 6) minutes on average.

#### 2.2.2 Procedure

After consenting to participate in the study, participants performed a discrimination task and then completed several questionnaires on disgust sensitivity to body odors, health anxiety and perceived vulnerability to disease. Participants could withdraw their participation at any time in which case their data were not saved. The online procedure was programmed with *Psychopy 3.0* [33]. The discrimination task and questionnaires are described below.

#### 2.2.3 Discrimination between sick and healthy individuals

The task was to discriminate between sick and healthy facial composites. Participants were not informed that the facial photographs were composites rather than individual faces. They were instructed to look at a series of 20 composite faces presented one at a time, and to push one of two buttons to indicate whether each face depicted a sick or healthy individual. Within each sex, half of the participants were instructed to push the left-arrow button for healthy and the right-arrow for sick, while the other half were assigned the opposite pairing. The facial composites were displayed for 5 s, each in a random order. Participants could indicate their choice at any time (no time-out), including after the face had disappeared. Before the task, participants completed two practice trials with a male and a female healthy facial composite (order randomized) created from faces that were not used in the experiment.

#### 2.2.4 Disease-related questionnaires

After the identification task, participants completed several short questionnaires. The first questionnaire, the body odor disgust scale (BODS), is a 12-item self-report measure that assesses feelings of disgust towards body-related odors such as upper body sweat and urine, with two subscales: disgust for one’s own body odors (internal sources) and those of other people (external sources) [34]. Participants rated the extent to which each scenario elicited disgust on a Likert type of scale ranging from 1 (not disgusting at all) to 5 (extremely disgusting). The second questionnaire, perceived vulnerability to disease (PVD), is a 15-item self-report tool assessing individuals’ concerns of disease transmission with two subscales: the germ subscale, which targets discomfort in situations with potential transmission of pathogens, and the infectability subscale, which measures perceived susceptibility to infectious diseases [35]. Because the last item of the questionnaire is outdated (‘I avoid using public telephones because of the risk that I may catch something from the previous user’), it was replaced with ‘I avoid using public telephones or grabbing handrails in public transport because of the risk that I may catch something from the previous user’. Participants responded to each item on a scale ranging from 1 (strongly disagree) to 7 (strongly agree). The third questionnaire used was the 14-item short health anxiety inventory (SHAI), which measures clinical health anxiety [36]. Each of the 14 items consists of a group of four statements that are scored from 0 to 3. Participants had to select the statement that described their feelings the best. For each questionnaire, an individual score was calculated as the mean across items. Finally, they were asked to rate their health on a scale ranging from 1 (very bad) to 5 (very good). Covid-related questions were also asked, but these questions were not analyzed in the current study.

#### 2.2.5 Exclusion criteria

We included a control question in both the BODS and PVD questionnaires to confirm that the participants were following the instructions and not giving random responses. For the control question in the BODS questionnaire, participants were instructed to not click on the scale but rather on a small cross displayed at the bottom of the screen. For the control question in the PVD questionnaire, participants were instructed to select the answer ‘strongly agree’. Participants who responded incorrectly on any of the control questions were excluded (*n* = 32).

### 2.3. Statistical analyses

All analyses follow the preregistered analysis plan unless otherwise specified. Descriptive statistics including means, SD and t-test results comparing men’s and women’s reaction time, self-reported health, BODS, PVD and SHAI are displayed in Supplementary Table S1.

#### 2.3.1 Discrimination between sick and healthy individuals

To determine whether human observers could discriminate between sick and healthy individuals based on early facial cues of sickness, we first assessed discrimination accuracy, using the method of signal detection. We calculated both the discriminability (*d*’), from the probability of true sickness detections and false alarm, and the decision criterion (*c*), for each participant (package *psycho* in R). While the discriminability *d*’ indicates how well sick and healthy faces can be discriminated (with *d*’ = 0 indicating no discrimination at all [random choice]) and higher values indicating an increased ability, the decision criterion reflects how biased the participant is from a liberal (*c* < 0, most of the faces are considered sick) to a conservative bias (*c* > 0, few faces are considered sick). We used a one-sided one-sample Wilcoxon signed rank test (instead of the preregistered one-sided one-sample Student t-test to account for the nongaussian distribution of the data) to examine whether the mean of the discriminability *d*’ was significantly above 0.

We then examined whether sickness condition improved participants’ ability to discriminate between sick and healthy facial composites by using a generalized linear mixed model (GLMM) with a binomial error structure (*glmer* function of the *lme4* R package). Our dependent variable was the decision made by a participant (0 when they answered that the face was healthy, 1 when answering sick) for each of the 20 facial composites. Our explanatory variable, sickness condition, represented the two categories of the composite (healthy vs. sick). We included a random intercept for each participant’s and facial composite’s ID, and random slopes for condition by participant and composite.

#### 2.3.2 Sex difference and the perception of sickness

To determine whether women overestimate the presence of sickness cues (i.e. classified more faces as sick) compared to men, we used a one-sided Wilcoxon rank sum test with continuity correction (instead of the preregistered one-sided Student t-test to account for the nongaussian distribution of the data) testing whether women’s decision criterion *c* was significantly lower than men’s decision criterion.

We also performed exploratory analyses examining potential sex differences in the accuracy of discrimination between sick and healthy facial composites. We first used two-sided Wilcoxon rank sum tests with continuity correction comparing the percentage of correct identifications and the discriminability (*d*’) between men and women.

Given that sex differences related to age, reaction time, general health, or disease-related personality traits, might potentially account for the higher sickness discrimination rate in women, we controlled for the robustness of the results by conducting multiple linear regression analyses. In these analyses, we used *c*, *d*’ and the percentage of correct identifications as dependent variables, while including sex as an exploratory variable and age, reaction time, self-rated health, PVD, BODS, and SHAI as confounding variables. Because these variables may be intercorrelated, we checked for multicollinearity among the confounding variables by calculating the variance inflation factor (VIF). However, we found no evidence of multicollinearity, as all variables showed a low VIF value for all models (VIF < 1.38).

Finally, we used a hierarchical drift diffusion modeling (HDDM) approach. One of the advantages of this approach is that it allows for the combination of both reaction times and accuracy into one parameter—drift rate (*v*). The drift rate reflects how fast a person can accumulate information that leads to a correct decision. Higher *v* values reflect better performance in a shorter time. We built a model using sickness condition (healthy vs. sick facial composites), participants’ sex and the interaction between sex and condition as exploratory variables. Using the *hddm* Python package [37], we estimated a model in which the drift rate was specified to vary for men and women, separately for the sick and healthy facial composite conditions. We report the estimated drift rate *v* along with its corresponding 95% credibility intervals. We used a Markov Chain Monte Carlo sampling method to draw 10 000 posterior samples with a burn-in of 2000 samples and evaluated the model fit by visually inspecting the chain traceplots and autocorrelation plot for each model parameter (i.e. threshold/decision boundary a, the nondecision time constant *t*, and *v*). Additionally, we reran the same model five times with chains of 2000 samples (500 burn-in) to obtain and compare the Gelman-Rubin statistic, which allowed us to judge chain stability [38].

#### 2.3.3 Disease-related traits and perception of sickness cues

To investigate whether participants with poorer health, who are more sensitive to disgust, vulnerable to disease and/or concerned about their health, overestimate the presence of sickness cues (i.e. have a more liberal bias), we used a linear regression examining whether a participant’s decision criterion (*c*) was negatively associated with self-reported health and the score for each of the three questionnaires (BODS, PVD, and SHAI) and their subscales. Because women self-reported higher scores than men on all the questionnaires [34, 35], we controlled for the robustness of the results by re-running the same models with the participant’s sex as confounding variable.

Statistical analyses were performed with R, version 4.1.3 [39]. For the linear regressions and GLMMs, the statistical significance of each variable was tested with likelihood ratio tests comparing the full model to those without the term of interest.

## 3. RESULTS

### 3.1 Discrimination between sick and healthy individuals

**Figure 2.**
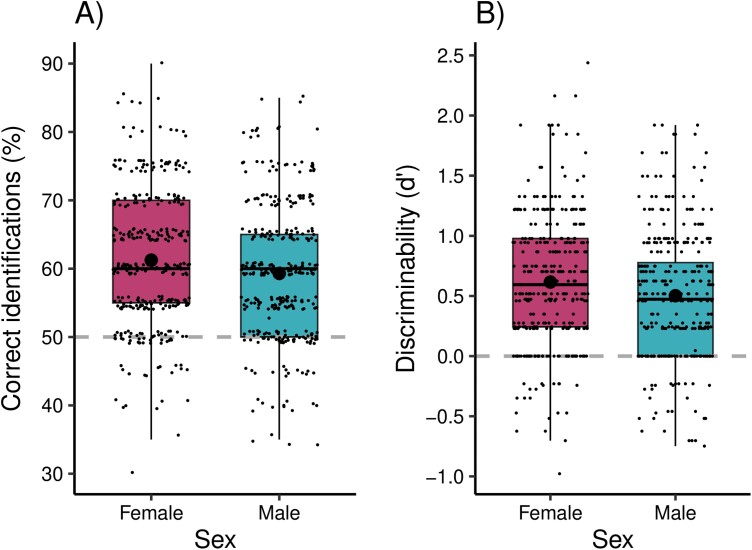
Women’s and men’s percentage of correct identifications (A) and discriminability *d*’ (B). Medians (thick lines), means (large black dot), first and third quartiles, whiskers extending to the largest and the smallest values no further than 1.5 times the inter-quartile range, and individual data points (small dots) are indicated. Grey dashed lines depict random choice.

In line with our prediction, human observers could discriminate between sick and healthy facial composites; on average, participants correctly identified 60.3 ± 9.9% of the faces (chance level 50%, Fig. 2A), and the discriminability *d*’ was significantly above zero (*d*’ = 0.56 ± 0.53; one-sided Wilcoxon signed rank test with continuity correction: *V* = 160 662, *P* < 0.0001, effect size *r* = 0.76, Fig. 2B). Similarly, the GLMM indicated that condition significantly influenced participants’ decisions (β = 1.22, SE = 0.17, χ^2^ = 50.28, *df* = 1, *P* < 0.0001).

### 3.2 Sex differences

Contrary to our hypothesis, women did not report the presence of sick faces (decision criterion *c*, Mean ± SD = 0.42 ± 0.50) significantly more than men (0.40 ± 0.49; one-sided Wilcoxon rank sum test with continuity correction: *W* = 60538, *P* = 0.81 effect size *r* = 0.03). However, our exploratory analyses indicate that women were better than men at differentiating between sick and healthy faces, as indicated by both percentage of correct identifications (women = 61 ± 1%, men = 59 ± 1%, Fig. 2A) and discriminability *d*’ (women = 0.62 ± 0.5, men = 0.50 ± 0.52, Fig. 2B) being significantly higher for women (correct identifications: two-sided Wilcoxon rank sum test with continuity correction: *W* = 64878, *P* = 0.01, effect size *r* = 0.10; discriminability *d*’: *W* = 65517, *P* = 0.005, *r* = 0.11). These findings remained robust even when controlling for confounding variables such as age, BODS, PVD, SHAI , self-reported health and reaction time, with participants’ sex significantly influencing percentage of correct identifications (β = −0.02, SE = 0.01, *F* = 6.87, *df* = 1, *P* = 0.009, Supplementary Table S2) and discriminability *d*’ (β = −0.11, SE = 0.04, *F* = 7.58, *df* = 1, *P* = 0.006, Supplementary Table S2) but not decision criterion c (β = −0.01, SE = 0.04, *F* = 0.04, *df* = 1, *P* = 0.84, Supplementary Table S2).

**Figure 3.**
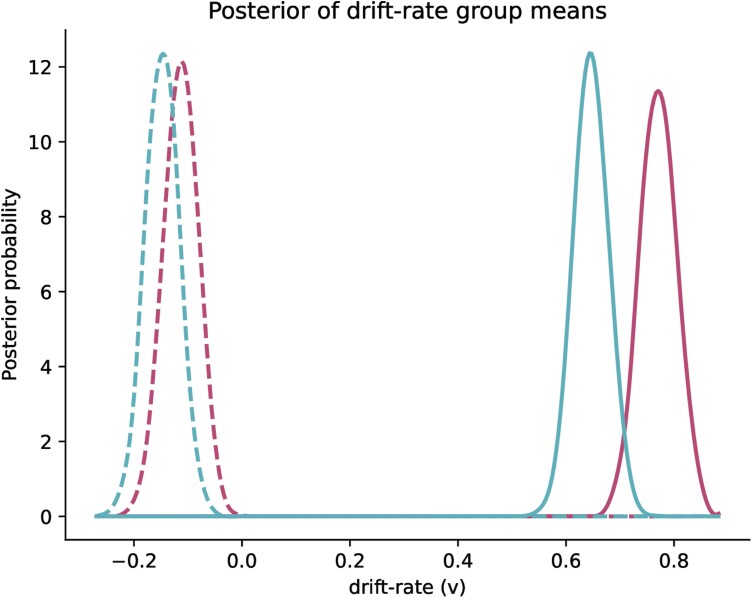
Posterior distributions for estimated drift rates (*v*) for men (turquoise) and women (red) in the sick (dashed lines) and healthy (solid lines) conditions. The peak of each distribution indicates most probable drift rate value according to our model. Higher drift rate values reflect more information accumulated per unit of time so that less time is needed to make a correct response.

In addition, the drift-diffusion analysis demonstrates that women were not only more accurate but were also faster than men at differentiating between sick and healthy faces (Fig. 3). Indeed, women exhibited higher drift rates, namely accumulated more evidence of sickness/healthiness per time unit, than men for both sick (women: *v =* −0.1 [−0.17, −0.04] vs. men: *v =* −0.14 [−0.2, −0.08]) and healthy faces (women: *v = *0.76 [0.7, 0.83] vs. men: *v* = 0.64 [0.58, 0.7]), with the probability of women having a higher drift rate than men being 0.78 for sick and 0.99 for healthy faces. Chains had symmetrical traceplots and low autocorrelation, and the R-hat Gelman–Rubin statistic was 1 ± 0.02 (Mean ± SD) indicating that the model converged well.

### 3.3 Influence of disease-related traits

**Figure 4.**
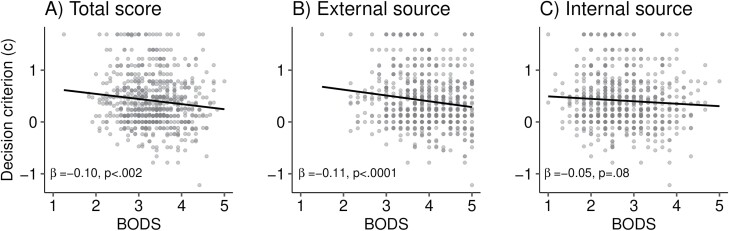
Scatterplots showing the relationship between the decision criterion *c* and the score on the BODS. The decision criterion reflects how biased a participant is from a liberal (*c* < 0, most of the faces are considered sick) to a conservative bias (*c* > 0, few faces are considered sick). Linear regressions showed that individuals who are more easily disgusted towards body odors (A), especially towards *others’* body odors (B), are more sensitive to potential sickness cues (i.e. have a lower decision criterion *c*). The decision criterion showed no statistically significant relationship with the internal body odor source subscale (C). Dots represent individual datapoints. The density of the data is illustrated by the intensity of the gray level of each data point.

Next, we tested whether participants self-reported health, disgust sensitivity towards body odors, vulnerability to disease and health anxiety were negatively associated with the decision criterion, a lower decision criterion reflecting a more liberal bias (i.e. most of the faces are considered sick). In line with our prediction, we found that individuals who are more easily disgusted by body odors, as measured by the BODS, are more sensitive to potential sickness cues (liberal bias), that is classify more faces as sick (β = −0.10, SE = 0.03, *F* = 10.12, *df* = 1, *P* < 0.002, Fig. 4A). This relationship seems mainly driven by disgust feelings towards *others’* body odors (i.e. external body odor source subscale, β = −0.11, SE = 0.03, *F* = 15.54, *df* = 1, *P* < 0.0001, Fig. 4B) compared to their own body odors (i.e. internal body odor source subscale, β = −0.05, SE = 0.03, *F* = 3.16, *df* = 1, *P* = 0.08, Fig. 4C). These results were robust to the inclusion of participant sex as a confounding variable—both disgust sensitivity towards body odors (BODS total score, β = −0.10, SE = 0.03, *F* = 10.24, *df* = 1, *P* < 0.002) and towards *others’* body odors (external body odor source sub-scale, β = −0.11, SE = 0.03, *F* = 15.88, *df* = 1, *P* < 0.0001) remained significantly and negatively related to the decision criterion, and the relationship between internal body odor source score and the decision criterion remained nonsignificant (β = −0.05, SE = 0.03, *F* = 3.14, *df* = 1, *P* = 0.08). Contrary to our prediction, however, a participant’s decision criterion was not significantly associated with self-reported health (β = 0.01, SE = 0.02, *F* = 0.42, *df* = 1, *P* = 0.52), PVD (total score: β = −0.02, SE = 0.02, *F* = 0.96, *df* = 1, *P* = 0.33; germ subscale: β = −0.003, SE = 0.02, *F* = 0.03, *df* = 1, *P* = 0.86; infectability subscale: β = −0.02, SE = 0.02, *F* = 1.57, *df* = 1, *P* = 0.21), nor with SHAI (β = −0.05, SE = 0.04, *F* = 1.42, *df* = 1, *P* = 0.23).

## 4. DISCUSSION

Because of the cost of sociality in term of transmission of infectious agents, it has been hypothesized that social animals evolved a complementary behavioral defense strategy [3, 4]. This prophylactic defense strategy includes the ability to accurately identify sickness cues, and a motivational response to avoid interactions with potentially infected individuals [4]. In fact, there is evidence that social species, including humans, identify sick conspecifics based on cues from different sensory modalities such as faces, body odors or biological motion [5–7, 10–12, 14]. Interestingly, humans do not only detect conspicuous cues of sickness (e.g. coughs, rashes, or sneezes) but also cues expressed only 2 h after the onset of an induced systemic inflammatory response [11–13]. Here, we replicate these findings by showing that our participants could discriminate between sick and healthy individuals based on facial composites, again supporting the view that humans are able to detect sickness cues only a couple of hours after exposure to an endotoxin stimulus.

The large sample of participants also provided enough power to investigate whether sensitivity to early facial sickness cues was associated with sex and disease-related personality traits. While we could not support the hypothesis that women have a more liberal decision criterion compared to men, exploratory analyses showed that women were faster and slightly better at discriminating between sick and healthy individuals. We hypothesized that the perception of sickness cues would differ between men and women, due to the evolutionary pressure on women to protect both themselves and their offspring from infectious disease [9] and because women exhibit substantially higher levels of disgust than men [23, 27, 40], including disgust towards pathogen connoting stimuli [9, 25, 26, 28, 34]. Better sickness detection and greater disgust sensitivity could enable women to reduce contaminations by triggering behavioral avoidance towards sick individuals. While the difference in discrimination accuracy between men and women is rather small in terms of effect size (*r* = 0.10), an HDDM approach showed that by combining both reaction times and accuracy, the likelihood of women performing better than men in a shorter period of time is actually over 78%. Besides, in our identification task, participants could base their decision through only one sensory cue. Hence, it still remains plausible that the sex difference may change—that is be greater or smaller—when individuals have the possibility of integrating sickness cues from different sensory modalities, such as body odors [11, 13, 20], voice [41] or gait [14]. In line with this, identification of sickness has been more accurate when visual and olfactory cues are presented simultaneously [12, 13]. Finally, a small sex difference in sickness detection could possibly be related to a greater sex difference in behavioral avoidance; thus, further investigations should explore whether sickness cues trigger behavioral avoidance and whether behavioral avoidance differs between men and women.

There are several alternative explanations for the demonstrated sex difference in ability to discriminate between healthy and sick faces. For example, women might have been more motivated than men to carry out the task to their best ability. However, such an effect would typically be accompanied by longer inspection time and thus manifest as a speed-accuracy trade-off. Instead, women were not only more accurate but also faster at responding. Differential exposure to sickness due to women’s higher parental care investment as compared to men [21, 22] could also lead to a better ability to discriminate between healthy and sick individuals through a learning process. In addition, many studies indicate that women outperform men in tasks related to face recognition and processing of facial emotional expressions [42, 43]. This higher ability to read facial expressions could be particularly important in identifying sick individuals as sick faces display more negative emotions such as sadness and disgust, and less happiness and surprise [16]. Therefore, it is possible that the higher accuracy of women in discriminating healthy from sick faces observed in our study stems from their better ability to identify facial expressions. Finally, another explanation could be the higher prevalence of color blindness in men (around 8% in Caucasian Europeans) as compared to women (0.4%) [44]. Previous studies found that skin coloration is an important facial cue of sickness: after endotoxin injection, facial skin become lighter and less red [15] and paleness of facial skin and lips were found to be associated with apparent sickness [10]. Although other cues, unrelated to skin color, were also associated with apparent sickness (such as having a more swollen face, droopier corners of the mouth, and more hanging eyelids) [10, 16], color blindness could impede the ability to discriminate sick from healthy faces. We estimated the potential influence of color blindness in our data set in an additional analysis where we simulated an inability to detect sickness due to color blindness in 8% of the females due to color blindness, matching the male prevalence. With this background, we randomly selected 7.6% (*n* = 26) of the females and replaced their decisions by random responses (50% sick and 50% healthy responses). In this conservative analysis, women’s *d*’ was still higher (*d*’ = 0.57) than men’s (*d*’ = 0.50; average of 1000 iterations), suggesting that color blindness is not likely to be the only factor explaining women’s higher accuracy. This exploratory result should nonetheless be replicated in a study controlling for color blindness.

In line with our prediction, we found that individuals who are more disgust sensitive to body odors were more prone to overestimate the presence of sickness cues and that this effect was specifically driven by disgust toward others’ body odors. However, no associations were found with PVD or health anxiety. It has been argued that disgust is an evolved motivational system for disease avoidance [40]. Indeed, pathogen-connoting stimuli trigger high levels of disgust [9, 25, 26, 28, 34]. The perception of others’ body odors indicates an interpersonal proximity in which pathogens have an increased chance of being transmitted, and diseases are known to affect the body odors of sick individuals including the smell of their sweat, urine, and breath [45]. High BODS scores are positively associated with disgust ratings of sweat odors suggesting that BODS is a valid marker of olfactory disgust [46]. Our finding that body odor disgust predicts the tendency to classify individuals as sick, but not discriminability, may be linked with previous research showing that individuals that are highly disgusted by body odors have more negative views of refugees [47, 48] and sympathize more with right-wing authoritarianism [49], two ideological dispositions that may restrict inter-group contact and social mobility. Hence, it could be speculated that high body odor disgust sensitivity arises from a dispositional bias that leans toward classifying other individuals as pathogen threats.

### 4.1 Study limitations

We used a database of early cues of sickness stimuli, collected during a study that used a within-subjects placebo-controlled experimental endotoxemia model, in the largest study on sickness detection performed thus far with almost 700 participants. However, our study presents a few limitations. First, our results are limited to visual detection and sickness stimuli that reflect a systemic inflammation (i.e. a nonspecific immune reaction to endotoxins). Akin to body odors for which specific smells may be associated with specific diseases [45], some diseases may be detected from different visual cues, a trivial example being chicken pox. Further studies are thus necessary to generalize our findings to different naturally occurring infectious diseases in a context more ecologically valid. Second, because of the COVID-19 pandemic, we conducted this experiment online rather than in a controlled laboratory environment. Although we used two quality check questions, it remained difficult to assess participants’ motivation to succeed at the task, as well as their honesty when completing the questionnaires. However, a recently published study comparing several recruitment platforms for online behavioral research found that *Prolific* provides high-quality data regarding participants’ attention, comprehension, honesty and reliability [50]. Third, conducting the study online forced us, due to ethical considerations, to use facial composites rather than individual faces. Facial composites were created by averaging real facial photographs; healthy composites were obtained by averaging individuals in a healthy state, and sick composites were obtained by averaging individuals who underwent an induced inflammatory sickness response. Although this method allows for creating realistic faces, we do not know if the participants realized that the faces were morphs (no participant commented on this aspect) and how this method might have affected the results. The discriminability index (*d*’ = 0.56) was, nevertheless, almost identical (*d*’ = 0.57 calculated based on their reported sensitivity [0.52] and specificity [0.30]) to that found in a previous study using the original photos [10], indicating that the relevant sickness cues were preserved. Furthermore, it is still unclear whether the ability to distinguish between sick and healthy individuals relies on recognizing specific facial cues of sickness, noticing the absence of certain facial cues of healthiness, or identifying general deviations in facial traits, such as emotional expressions or signs of fatigue, that are associated with sickness in the particular task used in the study. This aspect was not explored here, and it is still to be determined. Finally, it is important to note that our facial database of sickness cues is limited to Swedish participants, and the raters we recruited were only native English speakers, which limits the generalization of our findings to other cultures.

### 4.2 Conclusion

Our study provides support that humans can differentiate between sick and healthy individuals based solely on early facial cues of sickness and suggests that, while women did not overestimate the presence of sickness cues, women were significantly, albeit only slightly, better at discriminating between sick and healthy individuals than men. In addition to replicating this exploratory finding, future research should investigate whether women’s putative superior performance is related to increased avoidance of sick individuals and whether our findings generalize to naturally occurring contagious diseases as well as to other populations.

## Supplementary Material

eoad032_suppl_Supplementary_Figures_S1_Tables_S1-S2

## Data Availability

The data that support the findings of this study are openly available on OSF at https://doi.org/10.17605/OSF.IO/P85EZ.
