# Supplementary material for: Discriminating between sick and healthy faces based on early sickness cues: an exploratory analysis of sex differences
Source: Evol Med Public Health. 2023 Sep 27;11(1):386–96. doi: 10.1093/emph/eoad032 (PMC10629974; doi:10.1093/emph/eoad032)
Supplement: eoad032_suppl_Supplementary_Figures_S1_Tables_S1-S2 [file eoad032_suppl_supplementary_figures_s1_tables_s1-s2.docx]

**Discriminating between sick and healthy faces based on early sickness cues:**

**an exploratory analysis of sex differences**

**Tognetti et al., 2023 EMPH**

Supplementary Data

**Figure S1. Age distribution**

**
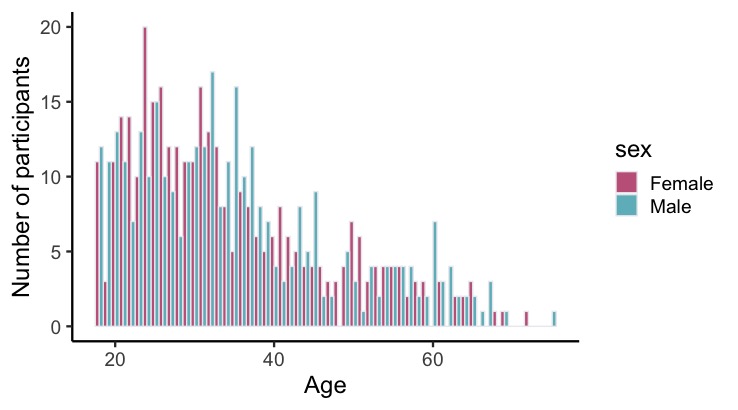
**

**Table S1. Descriptive statistics comparing men’s and women’s characteristics.** Mean and SD for men and women are given as well as the results of two-sided Student t-tests comparing men’s and women’s characteristics (except age). Variables that significantly differ between men and women are reported in bold.

|  |  | Men (N=340) | | Women (N=343) | | Two-sided Student t-test | | |
| --- | --- | --- | --- | --- | --- | --- | --- | --- |
|  |  | Mean | SD | Mean | SD | t | df | *P* |
| Age (years) | | 35.30 | 12.95 | 34.30 | 12.18 |  |  |  |
| **Reaction Time (s)** | | **1.74** | **0.92** | **1.54** | **0.77** | **-3.13** | **660.44** | **.002** |
| Self-reported health | | 3.82 | 0.85 | 3.78 | 0.84 | -5.17 | 680.86 | .61 |
| BODS | |  |  |  |  |  |  |  |
|  | Total score | 3.31 | 0.61 | 3.35 | 0.61 | 0.76 | 680.81 | .45 |
|  | External source | 3.86 | 0.67 | 3.94 | 0.65 | 1.51 | 680.22 | .13 |
|  | Internal source | 2.76 | 0.70 | 2.76 | 0.72 | -0.11 | 680.85 | .91 |
| **PVD** | |  |  |  |  |  |  |  |
|  | **Total score** | **3.85** | **0.88** | **4.20** | **0.98** | **4.95** | **673.9** | **<0.001** |
|  | **Germ subscale** | **4.21** | **1.17** | **4.41** | **1.15** | **2.29** | **680.5** | **.02** |
|  | **Infectability subscale** | **3.58** | **1.04** | **4.05** | **1.25** | **5.28** | **661.92** | **<0.001** |
| **SHAI** | | **1.84** | **0.43** | **1.96** | **0.45** | **3.48** | **679.11** | **<0.001** |

**Table S2.** **Multiple linear regression analyses investigating whether men (N=340) and women (N=343) differed in (A) decision criterion c, (B) discriminability index d’ and (C) correct identifications.** For each variable, the estimate (β), the standard error of the mean (SE), the F statistic, the degrees of freedom (df), and the p-value of the likelihood ratio of the comparison between the full model and the model without the factors are given. The estimate of the variable *Sex* is for the comparison between men and women (reference category).

|  | **ß** | ***SE*** | **F** | **df** | ***p*** |
| --- | --- | --- | --- | --- | --- |
| **A) Decision criteria c** |  |  |  |  |  |
| Intercept | 0.92 | 0.20 |  |  |  |
| Sex | -0.01 | 0.04 | 0.04 | 1 | 0.84 |
| Age | 0.01 | 0.01 | 0.58 | 1 | 0.45 |
| Response time | -0.11 | 0.02 | 24.52 | 1 | <0.001 |
| Self-reported health | 0.01 | 0.02 | 0.04 | 1 | 0.84 |
| BODS (total score) | -0.10 | 0.03 | 10.24 | 1 | 0.001 |
| PVD (total score) | 0.01 | 0.02 | 0.002 | 1 | 0.96 |
| HAI (total score) | -0.03 | 0.05 | 0.33 | 1 | 0.58 |
|  |  |  |  |  |  |
| **B) Discriminability index d’** |  |  |  |  |  |
| Intercept | 0.31 | 0.22 |  |  |  |
| Sex | -0.11 | 0.04 | 7.58 | 1 | 0.006 |
| Age | -0.01 | 0.01 | 2.84 | 1 | 0.09 |
| Response time | 0.08 | 0.02 | 10.70 | 1 | 0.001 |
| Self-reported health | 0.06 | 0.03 | 5.54 | 1 | 0.02 |
| BODS (total score) | -0.40 | 0.03 | 1.08 | 1 | 0.30 |
| PVD (total score) | 0.04 | 0.02 | 2.43 | 1 | 0.12 |
| HAI (total score) | 0.01 | 0.05 | 0.01 | 1 | 0.98 |
|  |  |  |  |  |  |
| **C) Correct identification** |  |  |  |  |  |
| Intercept | 0.56 | 0.04 |  |  |  |
| Sex | -0.02 | 0.01 | 6.87 | 1 | 0.009 |
| Age | -0.01 | 0.01 | 2.74 | 1 | 0.10 |
| Response time | 0.02 | 0.01 | 14.10 | 1 | 0.0001 |
| Self-reported health | 0.01 | 0.01 | 3.75 | 1 | 0.05 |
| BODS (total score) | -0.01 | 0.01 | 0.93 | 1 | 0.33 |
| PVD (total score) | 0.01 | 0.01 | 1.59 | 1 | 0.21 |
| HAI (total score) | 0.01 | 0.01 | 0.13 | 1 | 0.72 |
